# Molecular Dynamics Simulations of the Temperature Induced Unfolding of Crambin Follow the Arrhenius Equation.

**DOI:** 10.12688/f1000research.6831.1

**Published:** 2015-08-20

**Authors:** Andrew Dalby, Mohd Shahir Shamsir

**Affiliations:** 1 Faculty of Science and Technology, University of Westminster, London, W1W 6UW, UK; 2 Faculty of Biosciences and Medical Engineering, Universiti Teknologi Malaysia, Skudai, Johor, 81310, Malaysia

**Keywords:** Molecular dynamics, Arrhenius, unfolding rate

## Abstract

Molecular dynamics simulations have been used extensively to model the folding and unfolding of proteins. The rates of folding and unfolding should follow the Arrhenius equation over a limited range of temperatures. This study shows that molecular dynamic simulations of the unfolding of crambin between 500K and 560K do follow the Arrhenius equation. They also show that while there is a large amount of variation between the simulations the average values for the rate show a very high degree of correlation.

## Introduction

Molecular dynamics (MD) simulations have become an important tool in understanding chemical and biochemical processes at the molecular level. Through the use of Newtonian mechanics and an empirically derived force-field, simulations have been used to investigate the interactions of drug molecules and their targets as well as to predict the behaviour of proteins and peptides ( Vasquez *et al.*, 1994). Originally simulations were carried out *in vacuo*, but now with the increasing power of computers, simulations are usually carried out using periodic boundary conditions in water.

Increasingly realistic simulations lead to a better understanding of processes such as protein folding and unfolding at the molecular level ( Lindorff-Larsen *et al.*, 2012; Scheraga *et al.*, 2007). The literature on molecular dynamics simulations for protein folding and unfolding is extensive. This can include very large simulations that can be simplified using coarse-graining, down to simulations of small proteins or peptides. One area where MD simulations have been particularly widely used is in the simulation of prion proteins and the protein mis-folding diseases associated with them ( Shamsir & Dalby, 2005; Shamsir & Dalby, 2007; van der Kamp & Daggett, 2010; van der Kamp & Daggett, 2011).

Molecular dynamics simulations give us a detailed view of protein folding and unfolding pathways and their rates of unfolding ( Daggett, 2002). Temperature is often used to accelerate protein unfolding and it has been shown that this does not affect the protein unfolding pathway ( Day *et al.*, 2002). It is often difficult to relate the simulated results to experimental data. A review of simulated folding times has shown that the times predicted by MD and the experimental rates for thermal unfolding are in good agreement ( Snow *et al.*, 2005). Atomic force microscopy data is another possibility and this has been used to investigate protein folding of T4 lysozyme ( Peng & Li, 2008).

The rate of protein folding at increasing temperatures should be described by the Arrhenius equation over a limited range of temperatures (Alberty):

k=AeEaRT

Where k is the rate of the reaction, A is a constant (pre-exponential factor) E _a_ is the energy of activation, T is the Temperature in Kelvin and R is the Universal Gas Constant.

A rearrangement of the Arrhenius equation taking natural logarithms gives the linear function:

$$\ln(k)=\frac{-E_{a}}{R}\frac{1}{T}+\ln\;(A)$$

A plot of the natural logarithm of the rate ln(k) against 1/Temperature will be a straight line if the simulations obey the Arrhenius equation.

The Arrhenius equation has been used in solid-state chemistry calculations but currently no studies have tested whether it is valid in MD simulations of protein folding ( Huwe *et al.*, 1999). This paper presents a MD simulation study using a small protein (crambin) to test whether the models do agree with the predicted linear behaviour.

## Materials and methods

Crambin was chosen as the model protein for simulation of its size as it only has 46 amino acids ( Caves *et al.*, 1998). A high resolution crystal structure of crambin (3NIR.pdb) was downloaded from the RCSB Protein Databank ( Rose *et al.*, 2011; Schmidt *et al.*, 2011). Molecular dynamics simulations were carried out using Gromacs 4.6.4 on an Ubuntu 12.04 machine with GPU acceleration ( Hess *et al.*, 2008).

The protein was solvated using periodic boundary conditions and a surrounding distance of 4nm around the protein. Simulations were run at 500K to 560K at 10K intervals using the OPLS forcefield. At temperatures of 570K and above the simulations fail to complete. The models were initially equilibrated using the canonical (nvt) and isothermal-isobaric (npt) ensembles. At 500K the simulations were run for 10ns with a time-step of 2fs. At 560K the simulations were run for 1ns with a time-step of 2fs. Secondary structure was calculated using DSSP ( Dodge *et al.*, 1998) (the data is available in dssp_files.zip). RMSD deviations from the original crystal structure were calculated in Gromacs and displayed in Grace (the data is available in rmsd_grace_datafiles.zip). All of the scripts for equilibration dynamics and analysis can be found in the accompanying data files (the Gromacs files are in gromacs_files.zip and the shell script to run all the simulations and analysis is gromacs_runs_complete_analysis.sh).

The statistical analysis of the rate data was carried out using SPSS version 22 ( IBM_Corp., 2013). The line of best fit to the mean data was fitted using linear regression (the data files are available as md_arrhenius_crambin_averaged.sav, md_arrhenius_crambin_averaged.spv). The line of best fit for the complete dataset was calculated using the general linear model (the data files are available as md_arrhenius_crambin.sav, md_arrhenius_crambin.spv). All of the scripts and data for the statistical analysis are available in the supplementary materials.

## Results

**Figure 1.**
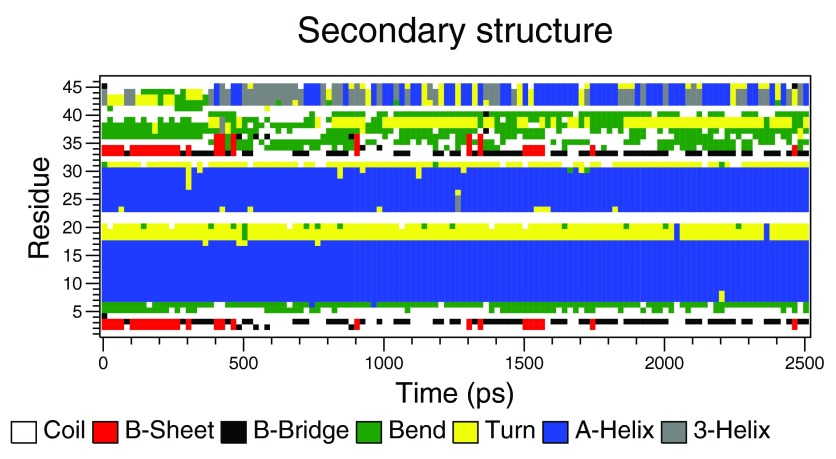
An Example DSSP Secondary Structure Plot (from 520K run 1).

Over these time periods crambin did not unfold as much as had been expected. As it is such a small protein it seems to be particularly stable to rises in temperature. There were three possible end points that could be used in determining the rate:

1) The unfolding of the C-terminal final bend ( Figure 1, the green region from residues 36-38).
2) The loss of the beta sheet ( Figure 1, the two red regions residues 2-4 and 33-34).
3) The increase of the root mean squared deviation (RMSD) to 0.4nm from the crystal structure.

**Figure 2.**
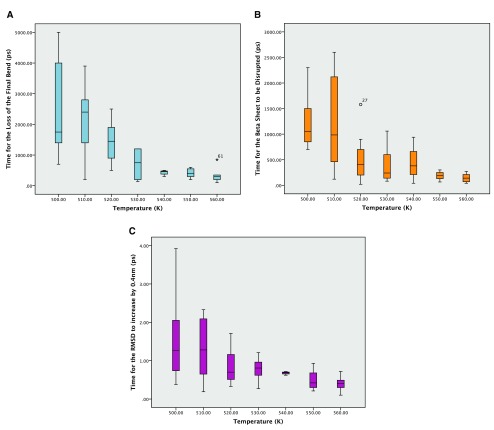
**A**: Boxplot of the times for the loss of the final bend from the secondary structure in picoseconds. Outliers are labelled. **B**: Boxplot of the times for the loss of the beta sheet in picoseconds. Outliers are labelled as circles or numbers if they are extreme. **C**: Boxplot of the times for the RMSD to go above 0.4nm from the crystal structure in picoseconds.

It was not clear which of the measures would be the most reliable and so time was taken for all three. There were however missing values because the end points were not reached during the simulations. This was particularly apparent in the 540K simulations, which seem to be anomalous as can be seen in the boxplots for the reaction times from the simulations ( Figure 2A–C). The boxplots show the expected downward trend, although there is considerable variation between the times taken for the repeated simulations. This variability declines at higher temperatures as rates become faster. A summary of the times to the three different end points are given in Table 1– Table 3.

**Table 1.** Times until the loss of the bend from residue 36-38 in the protein in picoseconds.

Temperature	Mean (ps)	Standard Error (ps)	95% Confidence Interval (ps)
500	2340	480	1252 to 3427
510	2190	341	1418 to 2961
520	1470	219	974 to 1965
530	679	142	357 to 1001
540	420	61	157 to 683
550	415	42	319 to 510
560	328	71	163 to 492

**Table 2.** Times for loss of the beta sheet from the protein in picoseconds.

Temperature	Mean (ps)	Standard Error (ps)	95% Confidence Interval (ps)
500	1235	163	865 to 1604
510	1195	274	575 to 1815
520	517	151	175 to 859
530	400	111	149 to 651
540	577	122	278 to 876
550	186	25	129 to 243
560	135	25	78 to 192

**Table 3.** Time for the RMSD to go above 0.4nm from the initial crystal structure in picoseconds.

Temperature	Mean (ps)	Standard Error (ps)	95% Confidence Interval (ps)
500	1563	361	746 to 2380
510	1313	253	741 to 1885
520	859	147	528 to 1190
530	774	101	544 to 1003
540	553	76	341 to 764
550	489	77	314 to 664
560	391	64	242 to 540

**Figure 3.**
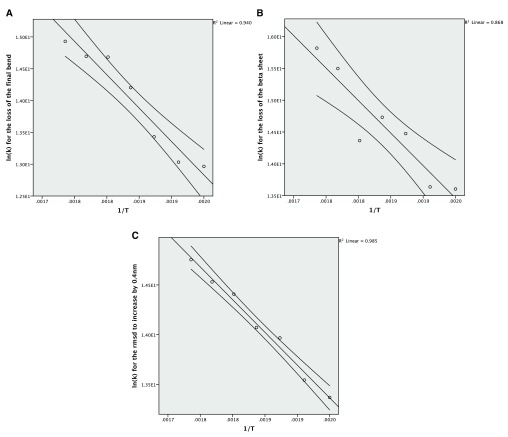
**A**: The Arrhenius plot for the unfolding of the final bend using the averaged data. **B**: The Arrhenius plot for the unfolding of the beta sheet using the averaged data. **C**: The Arrhenius plot for the increase of the RMSD by 0.4nm from the crystal structure using the averaged data.

**Figure 4.**
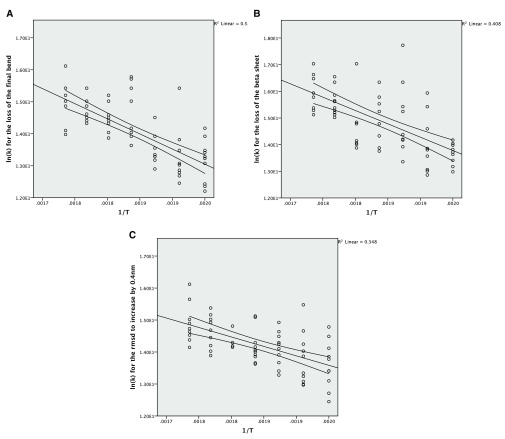
**A**: The Arrhenius plot for the unfolding of the final bend using the complete data. **B**: The Arrhenius plot for the unfolding of the beta sheet using the complete data. **C**: The Arrhenius plot for the increase of the RMSD by 0.4nm from the crystal structure using the complete data.

The Arrhenius plot can be constructed using either the mean values for the different temperatures or all of the values for the repeats. In both cases a linear model is produced but the correlation is much stronger for the averaged values, which also give a narrower confidence interval for the line of best fit ( Figure 3A–C). The wider confidence intervals for all of the data and the extent of the scatter can be seen in Figure 4A–C.

The parameters for the lines of best fit using all of the data and only the mean values are given in Table 4 and Table 5.

**Table 4.** Lines of best fit for the Arrhenius equation using all of the data.

End Point		Value	Confidence interval	Coefficient of determination
Bend loss	Gradient	-9458	-11760 to -7245	52%
	Intercept	32	27 to 36	
Beta sheet loss	Gradient	-10020	-13012 to -7027	41%
	Intercept	34	28 to 39	
RMSD > 0.4nm	Gradient	-5918	-7976 to -3860	35%
	Intercept	25	22 to 29	

**Table 5.** Lines of best fit for the Arrhenius equation using the mean rates.

End Point		Value	Confidence interval	Coefficient of determination
Bend loss	Gradient	-10500	-13555 to -7444	94%
	Intercept	34	28 to 40	
Beta sheet loss	Gradient	-10217	-14800 to -5635	87%
	Intercept	34	25 to 43	
RMSD > 0.4nm	Gradient	-6585	-7510 to -5660	98%

## Discussion

All three of the end points used produce a linear model in the Arrhenius plot with a high degree of correlation. This suggests that in these cases the MD simulations are following Arrhenius behaviour and that these models can be used to predict rates of unfolding.

Of the three end points the RMSD variation is the easiest to calculate and least ambiguous, but it is also difficult to understand what this signifies at the protein level, when compared to using the disruption of secondary structure as a metric. The other advantage of using the unfolding of the secondary structure is that experimental values are available for unfolding of proteins and so if an appropriate end point can be found in the simulations then the gradients of the Arrhenius plot can be used to calculate the activation energy for unfolding.

This study also highlights the high degree of variability between the trajectories of different simulations. This variability is very high at lower temperatures. Simulations were repeated ten times and this resulted in values for the standard errors for the time of unfolding that could be up to 25% of the time. These standard errors are large and so the number of simulations that are run needs to be increased in order to reduce them. As the standard errors falls with the square root of the sample size this would mean a 4-fold increase in the number of simulations is needed to reduce the standard error by half. This would suggest that more reliable results could be obtained by carrying out 40 simulations at each of the temperatures, which is a considerable additional computational burden.

The large amount of variation also affected the quality of the lines of best fit to the Arrhenius equation. The coefficient of determination was much lower when considering all of the data but nonetheless there is clear evidence of the simulations following Arrhenius behaviour. The confidence intervals for the linear models using all the data and the average data are similar. The variation in the gradient is too large for making comparisons with experimentally derived energies of unfolding and this is another reason why a larger number of simulations will be needed in future studies.

There was a surprising degree of agreement in the slopes and intercepts of the bend loss and beta sheet loss end-points. This suggests that the energies involved in stabilising the beta sheet and final bend are similar. This consistency is encouraging and suggests that detailed energy predictions will be possible from MD simulations.

Crambin was not an ideal case for using in this study. Although it is very small and allows the simulations to be run in a shorter time, the protein does not unfold very much and so longer time-scales are needed within the simulations. Prion protein is another possible model that could be used to test unfolding as this has been shown to unfold over short simulation times (< 10ns) ( Shamsir & Dalby, 2005). The other alternative is longer simulations times in order to produce clearer and less ambiguous end-points for the simulations.

## Data and software availability

All of the code and data required for carrying out the simulations is available from http://dx.doi.org/10.5281/zenodo.20550. The shell script used to perform the simulations is available from http://dx.doi.org/10.5281/zenodo.20544. The dssp plots and the RMSD graphs for the simulations are available from http://dx.doi.org/10.5281/zenodo.20548. The SPSS data files and output files that include the details of how the analysis was carried out are available from http://dx.doi.org/10.5281/zenodo.20549. The software is released under a MIT license.
