# Supplementary material for: Molecular Dynamics Simulations of the Temperature Induced Unfolding of Crambin Follow the Arrhenius Equation
Source: F1000Res. 2015 Aug 20;4:589. [Version 1] doi: 10.12688/f1000research.6831.1 (PMC4629273; doi:10.12688/f1000research.6831.1)
Supplement: Peer review history

# Peer review: Referee response for version 1

**DOI:** 10.5256/f1000research.7345.r10967

**Published:** 2015-10-28

**Authors:** Adrian Mulholland

**Affiliations:** 1 Centre for Computational Chemistry, University of Bristol, Bristol, UK

I've looked at this paper and have some comments and concerns, some of which reflect the other reviewer's comments.  

I believe that there have been previous demonstrations of Arrhenius behaviour in unfolding simulations, including for crambin (see e.g. Ferrara *et al*. J. Phys. Chem. B 2000, 104, 5000-5010) and other proteins (e.g. Piana *et al.* PNAS 109 17845–17850, doi: 10.1073/pnas.1201811109).

Consideration of protein stability is crucial here (see Scalley and Baker Proc Natl Acad Sci U S A. 1997 Sep 30; 94(20): 10636–10640) and as the current simulations are run at high temperatures where the protein is unstable, this is an issue. The dielectric behaviour of the water model at these high temperatures is also a concern for me.

I have read this submission. I believe that I have an appropriate level of expertise to confirm that it is of an acceptable scientific standard, however I have significant reservations, as outlined above.

# Peer review: Referee response for version 1

**DOI:** 10.5256/f1000research.7345.r10438

**Published:** 2015-10-14

**Authors:** Dmitry Nerukh

**Affiliations:** 1 Institute of Systems Analytics, Aston University, Birmingham, UK

I have two main critical points:

1. The conceptual one is that the loss of the beta sheet or the bend could not be, strictly speaking, classified as "unfolding".  There is very little in the text that describes how specifically the authors quantified the moment of this loss of structure. Looking at Fig. 1 it can be seen that both motifs come back for some periods of time.  Is this a loss of structure or just equilibrium fluctuations?  If the latter, then there are no observable unfolding event in the data.
2. As authors rightfully admit, the uncertainty of these loss of structure times is very high, especially at low temperatures.  This is most likely because the length of simulations, 1-10ns, is too short (as again, the authors mention this in the text) and also because the algorithm of determining these times is not statistically robust.  For proteins, even small ones like crambin, typical time scales for structural rearrangements is of the order of tens of nanoseconds.  Even dialanin takes hundreds of nanoseconds to accumulate statistically sound number of its very simple conformational transitions.

Taking into account these points, the main conclusion of the authors based on data shown on Fig. 2-4 appears unsubstantiated to me.

Overall, the paper is interesting and worth indexing if the authors more rigorously calculate the "loss of structure" times.  I would consider building a statistical network on the major conformational states, in the spirit of Markov States Model.  Then, the average transition times would faithfully represent the "unfolding" events.

I have read this submission. I believe that I have an appropriate level of expertise to confirm that it is of an acceptable scientific standard, however I have significant reservations, as outlined above.

# Peer review: Referee response for version 1

**DOI:** 10.5256/f1000research.7345.r10439

**Published:** 2015-09-28

**Authors:** Melchor Sanchez-Martinez

**Affiliations:** 1 Mind the Byte, Barcelona, Spain

The research article entitled 'Molecular Dynamics Simulations of the Temperature Induced Unfolding of Crambin Follow the Arrhenius Equation' by Dalby and Shamshir shows how the Molecular Dynamics simulations of crambin unfolding do follow the Arrhenius equation. It is addressing an interesting point as that Molecular Dynamics simulations of protein folding is used to exhibiting an non-Arrhenius behaviour and protein unfolding follow an Arrhenius equation, it is widely accepted. Testing if it is true is a really nice and interesting work.

In the article, there is a comprehensive explanation of study design, methods and analysis. The conclusions are well explained and justified on the basis of the results, Furthermore, the authors have made the data fully available, including the scripts to reproduce their work, being able to be revised by the author. That point makes the work more trustable and robust.

As a consequence of that, the manuscript is recommended to be approved. However there are some minor changes and comments that the authors may consider to improving the study.

The authors stated that "The Arrhenius equation has been used in solid-state chemistry calculations but currently no studies have tested whether it is valid in MD simulations of protein folding".  This phrase should be rewritten and clarified, because as it is written, it seems that this is the first time that the Arrhenius equation is used in protein folding simulations and that's not true because its usage is widely established in protein folding.

To gain statistical significance, the authors performed 10 replicas, which is a good number. However more replicas changing the force-field, for instance using Amberff99SB-ILDN, would be a good option to gain more statistics and at the same time ensure to avoid the force-field dependence of the results, as in folding/unfolding processes it is very relevant (see for instance Lindorff-Larsen *et al*., 2012). Simulations with one or two more force-fields, performing 10 replicas for each one, would be appreciated.

To explore if the protein is unfolded or not, three different metrics were employed. One of them  is the increase of the root mean squared deviation (RMSD) to 0.4nm from the crystal structure. Why 0.4 and not another value? There are some references to that in the literature or it is just an observed trend in the simulations? If it is an observed trend, would change varying the force-field. In Shimada *et al.*, 2001, they identified important structural regions of Crambin. Some of them are used by the authors as unfolding indicators, although the aminoacid numbers are not exactly the same. Thus, I wondering if helix 1 (residues 6-18,sequence: SIVARSNFNVCRL) could be also a good indicator of Crambin unfolding with the employed PDB structure, and if it is, if there is a reason to not be used.

As the authors stated longer simulations of 40ns or even longer should show a more realistic and picture of Crambin unfolding. It is true that it constitutes a considerable additional computational time, but will probably result in stronger and more sound results. Thus I encourage the authors to do it, at least, in the future.

I have read this submission. I believe that I have an appropriate level of expertise to confirm that it is of an acceptable scientific standard.

# Response

**Published:** 2015-09-30

**Authors:** Andrew Dalby

**Affiliations:** University of Westminster

I would like to thank the referee for his helpful comments.

We definitely need to correct that omission in referring to other work using Arrhenius on proteins, although it has mostly been on short segments of protein or peptides rather than a complete protein. In writing the introduction I was surprised by the lack of literature on using Arrhenius in a biological simulation context, except for enzyme simulations. I had searched for Arrhenius behaviour in molecular dynamics and as stated I only found references to its use in simulations of materials. It seems that the problem was I should have searched just for Arrhenius and in using behaviour it needs to be the US spelling, behavior to return the protein modelling literature! The work of Baker, Karplus and Kuriyan, and Pande are particularly important in establishing the field. The more recent work by Best and Mittal is another excellent example which incorporates the effects of forcefields.

I agree with the point about the forcefield but this adds a confounding variables and also increases the variance of the simulations, which increases the uncertainty in the gradient and has a negative impact on the confidence intervals for predicted energy barriers. An alternative is to use the technique to calculate relative differences in energy barriers between protein variants using the same forcefield. As you are calculating a difference the forcefield effects can be considered to cancel out. This was the idea behind free energy perturbation calculations.
